# Overcoming Challenges in Video-Based Health Monitoring: Real-World Implementation, Ethics, and Data Considerations

**DOI:** 10.3390/s25051357

**Published:** 2025-02-22

**Authors:** Simão Ferreira, Catarina Marinheiro, Catarina Mateus, Pedro Pereira Rodrigues, Matilde A. Rodrigues, Nuno Rocha

**Affiliations:** 1 RISE-Health, Center for Translational Health and Medical Biotechnology Research (TBIO), ESS, Polytechnic of Porto, R. Dr. António Bernardino de Almeida, 400, 4200-072 Porto, Portugal; cms@ess.ipp.pt (C.M.); mar@ess.ipp.pt (M.A.R.); 2 Centro Hospitalar de Vila Nova de Gaia/Espinho, 4430-999 Vila Nova de Gaia, Portugal; s-cmarinheiro@ucp.pt; 3 Faculdade de Ciências da Saúde e Enfermagem, Universidade Católica Portuguesa, 1649-023 Lisboa, Portugal; 4 MEDCIDS—Department of Community Medicine, Information and Decision Sciences, Faculty of Medicine, University of Porto, 4200-450 Porto, Portugal; pprodrigues@med.up.pt; 5 CINTESIS@RISE—Centre for Health Technologies and Services Research, 4200-450 Porto, Portugal

**Keywords:** monitoring, physiological, artificial intelligence, industry, healthcare, video assisted techniques, heart rate monitoring, recommendations, health planning, real-time systems

## Abstract

In the context of evolving healthcare technologies, this study investigates the application of AI and machine learning in video-based health monitoring systems, focusing on the challenges and potential of implementing such systems in real-world scenarios, specifically for knowledge workers. The research underscores the criticality of addressing technological, ethical, and practical hurdles in deploying these systems outside controlled laboratory environments. Methodologically, the study spanned three months and employed advanced facial recognition technology embedded in participants’ computing devices to collect physiological metrics such as heart rate, blinking frequency, and emotional states, thereby contributing to a stress detection dataset. This approach ensured data privacy and aligns with ethical standards. The results reveal significant challenges in data collection and processing, including biases in video datasets, the need for high-resolution videos, and the complexities of maintaining data quality and consistency, with 42% (after adjustments) of data lost. In conclusion, this research emphasizes the necessity for rigorous, ethical, and technologically adapted methodologies to fully realize the benefits of these systems in diverse healthcare contexts.

## 1. Introduction

In an era where integrating technology seamlessly into daily life is crucial, the healthcare sector is on the brink of a major transformation [1]. The pressing need for unobtrusive, continuous health monitoring methods is more evident than ever, especially in scenarios where conventional monitoring techniques fall short [2].

As global healthcare organizations harness artificial intelligence (AI) and machine learning (ML) to forge new paths in treating critical ailments and enhancing care, the potential of video-based systems in navigating health-monitoring complexities emerges, demanding a nuanced understanding of technological, ethical, and practical obstacles [3]. Leveraging video versatility, the evolution in mobile-assisted health monitoring marks a paradigm shifts towards more accurate, comprehensive, and responsive health monitoring systems significantly enhanced by machine learning algorithms for a quick and approachable health assessment [4].

Remote monitoring or telehealth, boosted by AI and ML, has experienced a significant surge, particularly during the COVID-19 pandemic, offering advanced patient monitoring and diagnostic capabilities [5]. A recent review of remote monitoring systems including video states that this method can be as effective, or even more so, than traditional care, but emphasizes the need for more extensive studies across various fields to fully understand its clinical effectiveness in diverse contexts [6]. However, the full potential of video monitoring remains largely untapped, with the majority of research focusing on video consultations rather than comprehensive video monitoring [7]. Recently Shaik and his team [8] mentioned that ML and image processing techniques have been essential in telehealth monitoring, enabling the supervision of vital signs such as heart rate, respiratory rate, and blood pressure, citing studies by Rohmetra et al. [9], Bousefsaf et al. [10], Cho et al. [11], and Khalid et al. [12] that illustrate the effectiveness of these AI methods, including the use of convolutional neural networks for stress level analysis and remote photoplethysmography (rPPG) [13]. They even went further and mentioned facial and emotion recognition in studies like those of Mahesh et al. [14], Zainuddin et al. [15], Chowdary et al. [16], and Q. Xu et al. [17], achieving significant accuracy in classifying various emotional states. Although the results from video monitoring are promising and offer compelling evidence, its application remains limited in various fields, particularly in uncontrolled environments. Recent studies have shown promising results, with 67% accuracy in unsupervised learning and up to 82% with labeled data [18]. This leads to uncertainty about its true accuracy rates. In contrast, in laboratory settings or highly controlled environments, video analysis is often tailored or heavily regulated. This paper aims to change the current paradigm by presenting the real-world challenges of implementing video monitoring in a specific scenario: monitoring knowledge workers. In this study, we will begin by addressing challenges that are often overlooked, including data collection, video data processing and, most importantly, ethical considerations. Finally, we consider opportunities in video-based health monitoring and future directions, and offer concluding remarks.

### 1.1. Challenges in Data Collection

While using video-monitoring, it is paramount to have a camera setup that can leverage data collection in a suitable manner [19]. Training a computer vision algorithm is inherently reliant on the availability of data and the balance between model complexity and computational efficiency [20,21]; however, video datasets often contain various biases and limitations [22]. Additionally, assembling a dataset from scratch is frequently a costly endeavor, requiring high-resolution videos and substantial data quantities to effectively train specific features [23,24,25,26]. It is also very time consuming, since real-world video data collection cannot be accelerated [26]. Also, most commonly, video datasets carry unexpected biases, since they cannot be trained in every situation or outcome [27,28,29]. A well-known paper on computer vision presents this same bias in a simple situation, showing autonomous vehicles’ vision systems are better at spotting light-skinned pedestrians. This can result in fatal outcomes if they fail to equally detect diverse skin colors [30].

Furthermore, what about video monitoring with digital cameras or webcams? To enhance rPPG and motion-based respiratory signal quality and expand the camera-based usability, even amid non-physiological movements (such as during sports), a focus on reducing motion artifacts and noise cancellation is crucial [31,32].

When working with video monitoring using webcams, as in our scenario, numerous challenges are anticipated in data collection. Firstly, a computer can only utilize the webcam video feed for one function at a time. This limitation becomes more problematic considering the significant rise in video conferencing calls following the COVID-19 pandemic, which makes the task of continuous health monitoring using webcams impractical. Another challenge arises from movement or motion artifacts, as real-world data show that workers are seated for 79% of their work hours [33]. Contrasting this, the shift to working from home has led individuals to spend about 89% of their time seated, marking a notable 10% rise compared to office settings [34].

Moreover, many businesses provide their staff involved in less demanding office tasks with computers of lower performance, potentially hindering the efficient operation of local models on these devices. Ethical data collection dictates that each participant must initiate and terminate video data collection, highlighting the crucial role of human intervention in completing the process [35]. Additionally, it is widely recognized that motivating a large number of people to actively contribute to data collection remains a significant challenge.

### 1.2. Challenges in Processing Video Data

Video data present lots of challenges, some of those being volume, storage, data variety, velocity of data generation, time-consuming analysis, surveillance and privacy concerns, and the complexity of filtering data to determine what should be erased [36,37,38,39,40,41].

A notable challenge lies in the high computational requirements essential for processing the intricate nature of video data [42]. This issue is further exacerbated by the necessity for effective video compression and streaming techniques, which are crucial for efficient data management [43,44]. Moreover, the variability in video quality—affected by different angles, lighting conditions, and skin pigmentation—complicates the task of ensuring consistent data quality [45,46].

Maintaining both the quality and consistency of video data is a critical yet daunting task [47,48]. The challenge is further amplified when dealing with vast video datasets, which demand consistent data labeling. This process requires diligent care and the adoption of standardized methodologies [49]. Finally, the collection of specific health-related data often requires specialized equipment, such as infrared cameras for thermal readings or devices for precise pupil detection. These necessities introduce additional layers of complexity and expense to the video data processing landscape [50,51]. Recent advancements in video-based health monitoring have emphasized the integration of AI with sensor–cloud architectures to improve decision support in remote healthcare [52]. Additionally, research on low-cost sensor networks has demonstrated the feasibility of AI-driven vital sign monitoring for vulnerable populations such as the elderly, further supporting the scalability of video-based solutions in healthcare settings [53].

### 1.3. Ethical Challenges

Over the years we have seen that integration of information and communication technology in delivering health services has expanded significantly, creating a diverse range of applications. Have we explored the ethical challenges at the same pace? It is crucial to integrate ethical guidelines into video monitoring practices to adeptly manage these evolving circumstances. Carneiro and colleagues [54] have examined the benefits and drawbacks of innovative techniques for stress assessment and monitoring in the workplace. In their paper, the authors identify computer vision (or video-based monitoring) as the most accurate method but one facing challenges such as intrusiveness. Also, a 2021 systematic review [55] focusing on applying ethical principles in telehealth presented five main topics discussed in papers being: autonomy, beneficence, justice, nonmaleficence and professional–patient relationships. Despite being an interesting approach, the paradigm can be even harder in workplace monitoring, the main challenges being in sharing data, even in aggregated form to team managers or colleagues higher in the hierarchy chain. Also, the professional–patient relationships do not directly apply, being the main developing force directed to personal health monitoring and recommender systems [56,57]. When talking about data sharing, it is imperative to touch on the General Data Protection Regulation (GDPR) in the EU. In the realm of data protection legislation, health-related data refer to all personal details pertaining to an individual’s physical or mental well-being. This encompasses information that discloses the health condition of a person (referenced from Points 1 and 15, Article 4 of the GDPR). To be clearer, this is a very wide definition of “health data”: it can range from medical-related information (e.g., medical reports) up to non-medical content such as health data from wearables.

During the COVID-19 pandemic, the digital surge brought many different monitoring systems. However, these cannot be placed in the same category as health video monitoring. In fact, video monitoring even led to recommendations of future stress management after COVID-19 [58]. As wellness applications and wearable technologies become more prevalent, the practice of monitoring employee health is expected to expand, forming part of a broader trend of supervising employee productivity. We must clearly delineate between data that can be shared with employers and data that exclusively belong to employees. The GDPR establishes the foundational guidelines for handling employee data, emphasizing core principles and regulations. National laws and collective agreements are tasked with defining the boundaries for handling health data, implementing specific measures to safeguard employee dignity, legitimate interests, and fundamental rights. At the Council of Europe level, processing health data is advised only when absolutely necessary for specific job recruitment or to meet employment-related legal requirements, in line with national legislation and Convention 108+.

Health data collection is permissible under certain exceptional circumstances as defined by law. Point 2 of Article 9 of the GDPR specifies the conditions under which such data processing may occur, with explicit consent being one key exception. This form of consent is commonly utilized by health apps to collect, use, and distribute health data. In the context of employment, each EU member state’s national laws or collective agreements clearly state the conditions for obtaining consent, in accordance with Recital 155 of the GDPR.

However, the validity of explicit consent in the context of employee monitoring is contentious. According to Point 11, Article 4 of the GDPR, such consent must be given freely and without ambiguity. While employees often view workplace monitoring systems as a necessary compromise to enable remote work, the freedom of their consent is questionable. If consent is a prerequisite for entering a contract where data processing is not a necessity, then it cannot be considered freely given, as stipulated by Point 4, Article 7 of the GDPR.

With the evolution of workplaces towards remote work, digitalization, and cost reductions, monitoring employee health seems to be an enduring trend. Health data, being especially sensitive, demand careful handling when collected by employers. The design and usage of health tracking systems must adhere to data protection laws. Additionally, considerations extending beyond data protection warrant further scrutiny. It is yet to be determined whether current safeguards, primarily formulated for conventional workplaces, are adequate for the challenges posed by a digitalized work environment.

Even more recently, on 1 August 2024, the AI Act introduced strict regulations under Article 5(f) and Recital 44 concerning AI systems designed to infer emotions in workplace and educational settings. These provisions prohibit the placement on the market, deployment, or use of AI systems that attempt to analyze or infer emotions from individuals unless they serve strictly medical or safety purposes. This restriction come from serious concerns about the scientific reliability of such technologies, given that emotional expression varies across cultures, contexts, and individuals. The limited accuracy, lack of specificity, and poor generalizability of emotion recognition in AI raise risks of discriminatory outcomes and intrusions into fundamental rights. Particularly in work and education, where power imbalances already exist, the use of such systems could lead to unfair or detrimental treatment of certain individuals or groups. Therefore, the default prohibition of these AI systems seeks to prevent misuse, protect individual freedoms, and mitigate ethical risks, while allowing exceptions solely for applications related to healthcare and safety.

## 2. Pilot in Knowledge Workers

This study, extending over a three-month period, was dedicated to the acquisition of physiological metrics such as heart rate, blinking frequency, and emotional states to collect a stress detection dataset. It utilized advanced facial recognition technology embedded in the participants’ computing devices (software). The technology employed was adept at capturing these data through the device cameras, without the need for external data storage, thereby upholding the privacy of personal data. This approach was essential for improving adherence, ensuring thorough compliance with data governance protocols, and conducting comprehensive risk analysis, reflecting the outcomes of appropriate data management and risk assessment.

To preserve the anonymity of the study subjects, all the acquired data were encrypted and retained exclusively on the participants’ personal computing devices. The participants, as the holders of their personal data, were tasked with submitting these data to the research team, contingent upon their agreement. This study aligned with ethical norms as outlined in the Declaration of Helsinki and had the prior sanction of the Ethics Committee at the School of Health, Polytechnic Institute of Porto. All participants were thoroughly informed about the study’s objectives and methodology and consented digitally, affirming their agreement with a signature.

### 2.1. Participant Demographics

This investigation encompassed 46 participants, comprising 31 females and 15 males. To be eligible, participants were required to undertake computer-related activities for at least four hours daily. The average computer usage among the group was approximately 30 h weekly.

The study required participants to operate the webcam data collection application when working in front of the computer for more than one hour. They were also instructed to position themselves in front of the webcam for a minimum of four hours daily in a well-illuminated setting, to guarantee the accuracy and reliability of the data. Before starting the data collection phase, the participants were trained in the software operation, completing questionnaires, understanding data governance principles, and adhering to ergonomic standards for computer-based tasks.

### 2.2. Methods of Data Acquisition and Analysis

For the purpose of gathering data, software prepared for the project was employed. This software, operating through a webcam, captures physiological data such as blinking frequency, heart rate, and emotional indicators, all the while not capturing any visual footage. The collected data were stored on the participants’ own computers and were shared voluntarily, maintaining privacy and ownership. Key variables measured were emotional states, heart rate, and blinking frequency. The blinking rate was calculated using the number of blinks detected by the system, the frame rate of each participant’s computer, and the acquisition rate. The analysis entailed assessing each blink by the degree of eye closure. This software underwent comprehensive evaluation to ensure impact, and the data gathered were strictly used for academic research and collective dissemination purposes.

## 3. Results

In the pilot study, the mean age of participants was 44.78 (SD = 9.17) years with a standard deviation of 9.17. The actual recorded data amounted to 114,655.95 min with a standard deviation of 4498.31 min (more than 16.5 million data points), while the maximum expected duration of recorded data were 720,000 min, corresponding to a 6.27 ratio of expected to actual working hours with a daily variability of ±1.27 h.

**Figure 1:**
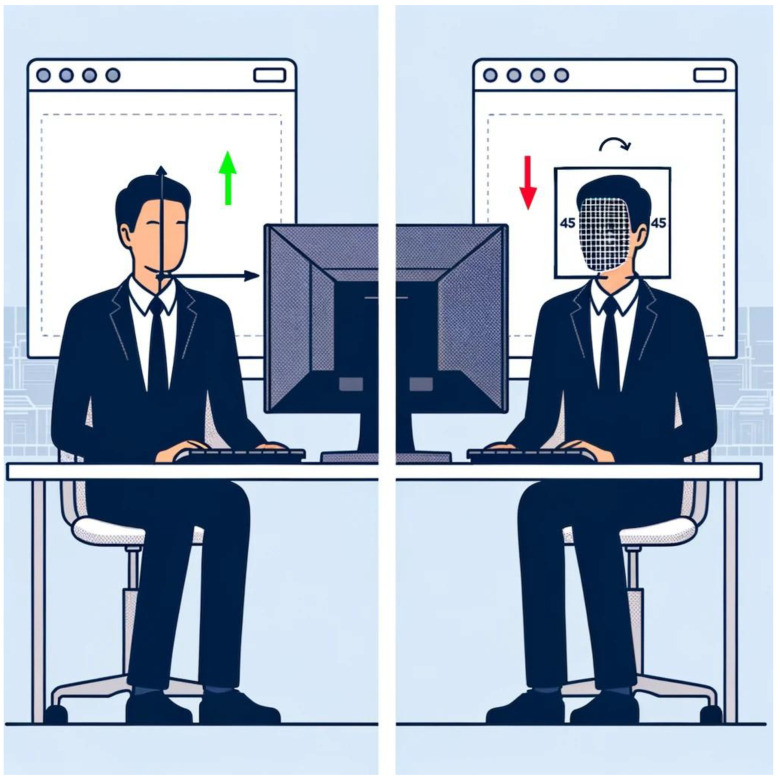
Illustration of reduced face detection efficacy in dual monitor work environments. (The arrows indicate efficacy levels: green for higher and red for lower).

All participants had consented to being monitored during their working hours. There was an approximate data loss of 84% relative to the maximum expected value. Nonetheless, when recalculating based on an estimated 4 h of active computer use per day, the percentage of data loss was reduced to 42%. While initially appearing substantial, this adjusted figure suggests a less severe impact on the study’s data collection efforts, considering the nature of the pilot study and the real-world constraints on participants’ available time. However, it was determined that the 114,655.95 min represent the cumulative time participants were actively engaged at their computers, uninterrupted and not occupied with activities such as Zoom meetings. Additionally, the data presented here have been filtered for optimal usability. They encompass instances where a face is detected with a confidence interval exceeding 60%. It is important to note that face detection efficacy decreases when participants are facing a colleague or engaged in other activities that do not involve being completely front-facing or within a 45-degree angle, as might be the case with double monitor usage (Figure 1). Consequently, the times provided represent actionable data.

In this pilot study on video monitoring, we encountered a dropout rate of 10%, equating to five actual dropouts. The final dataset was derived from 28 participants, attributable to various reasons. One participant left the company, and four did not respond to follow-up communications. Five participants experienced significant issues, primarily concerning synchronization with OneDrive. Two reported critical computer problems, necessitating a complete drive reformat. Six participants’ job responsibilities, involving extensive video conferencing, webcam-based software, or field work, were incompatible with video data collection. Additionally, four participants had major issues with corrupted files during data transfer; despite multiple days of data, all files were empty.

## 4. Discussion

This pilot study has highlighted several challenges and considerations that must be addressed to fully harness the potential of AI and ML in revolutionizing healthcare through remote monitoring technologies. Starting by tackling the discrepancy in data volume, these underscore a critical challenge in deploying video-based health monitoring systems in uncontrolled environments. However, it is important to recognize that without monitoring strategies, workplaces lack any means of generating health reports of any kind. Although our results reflect realistic user behavior, participants’ engagement time seemed to be considerably lower than anticipated. However, this may not be the case, bearing in mind the presented values were optimal data for feature extraction. Allowing some data to be available for feature extraction can permit a window into understanding and noting health status in the workplace. Video-based health monitoring offers a completely unobtrusive approach, with the distinct advantage of eliminating the need for smartwatches, smartbands, deployed sensors, or internet of things devices [59]. Of course this does not come without a price: when comparing video monitoring to other more traditional methods we must check advantages and disadvantages (Table 1).

The real impact of video-based health monitoring lies in its ability to provide continuous, unobtrusive physiological and behavioral tracking without requiring wearables or specialized clinical equipment. Unlike wearables, which require active user engagement, video-based systems passively analyze physiological signals such as blinking frequency and facial micro-expressions, enabling stress detection, fatigue analysis, and workplace well-being assessments in a scalable manner. This approach is particularly useful for knowledge workers and other sedentary populations where traditional monitoring solutions may be impractical or intrusive. While challenges such as lighting variability and data privacy must be addressed, AI-driven improvements in video-based systems continue to enhance their real-world applicability as a viable alternative to wearable and clinical monitoring solutions.

The ethical dimension of video monitoring was diligently considered in this study. With the established deferred line in place for initiating the pilot, our participants were volunteers who provided their signed consent directly to us (Research Center). The data collection was based on the principle that the participants were the sole owners of the collected data. At the conclusion of the pilot, the participants endorsed a comprehensive report to the company, acknowledging their role as facilitators in this pilot. However, these data merely represented the aggregated potential for managing and leveraging optimized workplaces. The privacy of the participants was preserved through encrypted data stored on personal devices, with voluntary submission to the research team. This approach, while ethical, introduced a dependency on participant compliance, which possibly contributed to partial data loss. Although there was data loss, explaining data ownership and data governance principles to the participants bolstered their confidence in the protocol and encouraged them to be more vocal in presenting data. Even though video cameras are regarded as one of the most privacy-sensitive methods [60], they present a unique window of opportunity that is seldom seen in pilot projects of any kind. While video monitoring is a specifically sensitive case in health data monitoring, these principles can be used in other pilots. After carefully drawing the line between personal health data and actionable/sharable data, we managed to make our participants completely at ease with data collection and the data cycle. Personal data can only be shared if the participant is willing to do so, bearing in mind the GDPR Point 11, Article 4 regarding consent [61].

Also, it was important to explain the data cycle to participants, making them aware of who was the data recipient and the purpose of data processing according to GDPR Point 9, Article 4.

Addressing the challenge of obtrusiveness, we considered the users’ perception regarding the need to manually toggle the application on and off for data collection. Simultaneously, while recognizing the advantages of unobtrusiveness, ethical principles impose limitations on completely unobtrusive and frictionless use of monitoring systems [62]. To enhance data retention and minimize user dependency, future iterations of the system could incorporate automated background data collection, reducing the need for manual activation. Additionally, adaptive sampling techniques and real-time integrity checks could be employed to ensure data completeness despite computational fluctuations. Cloud-assisted buffering and edge computing strategies may further help mitigate data loss by offloading processing tasks from low-performance devices, thereby improving consistency in video-based health monitoring.

Participant dropouts occurred at a rate of 10%, and there were additional participants from whom we could not analyze data due to previously mentioned reasons. It is vital to tackle these difficulties with video-monitoring tools, particularly in the context of post-COVID-19 work systems, where there has been a substantial increase in the reliance on video conferencing. This shift represents a significant challenge for continuous health monitoring. Bearing this in mind, the ideal setup would involve having at least two front-facing cameras continuously connected to the same computer. The specific ideal setup introduces yet another major challenge: with the evolving model of hybrid work [63], workers have different setups at home than in the office. Consequently, their home office setups need to be documented and reviewed to facilitate effective video data collection.

The limitations of computing hardware among participants, especially those provided by employers for light office work, emerged as another obstacle. This finding suggests that future implementations should consider the technical capabilities of participant hardware. Although our system was designed to run on any standard computer, participants using lower-performance devices reported noticeable slowdowns during monitoring. Further testing revealed that in resource-limited systems, the software experienced frame rate drops and processing delays, impacting data consistency. These findings indicate that hardware constraints can significantly influence the effectiveness of real-time video-based health monitoring, as lower frame rates reduce the number of analyzable frames, potentially affecting accuracy. Future optimizations should focus on adaptive algorithms that adjust processing demands based on available system resources, ensuring broader accessibility without compromising data quality. Furthermore, cloud-assisted edge computing could be leveraged to offload intensive processing tasks, thereby reducing the resource strain on low-performance devices while maintaining high data accuracy. While our study employed multiple data quality assessment indicators (post-collection), future implementations could integrate additional automated quality control metrics, such as signal-to-noise ratio (SNR), illumination consistency, and motion blur assessment, to further enhance data reliability. These metrics would provide deeper insight into external factors affecting facial recognition accuracy, particularly in varied lighting conditions and user movement scenarios. At the configuration time of the system, we had a confidence interval of only 60% for facial detection. This study’s findings align with broader literature indicating the efficacy of AI-enhanced video monitoring in health assessments [64]. However, our results also highlight the gap between controlled laboratory accuracy and real-world application [65]. To improve the generalizability of findings, future studies should incorporate a broader participant demographic, ensuring representation across different ethnic backgrounds, age groups, and occupational settings. This would enhance the robustness of models and reduce biases in physiological monitoring. Additionally, testing the system under varied environmental conditions, such as different lighting levels and camera angles, would further strengthen its applicability in real-world deployments.

This pilot study has provided valuable insights into the complexities of implementing video-based health monitoring in real-world settings. The lessons learned here will inform the design of more resilient and ethical remote health monitoring systems and guide future explorations into the promising intersection of AI, machine learning, and healthcare.

## 5. Opportunities in Video-Based Health Monitoring

**Figure 2:**
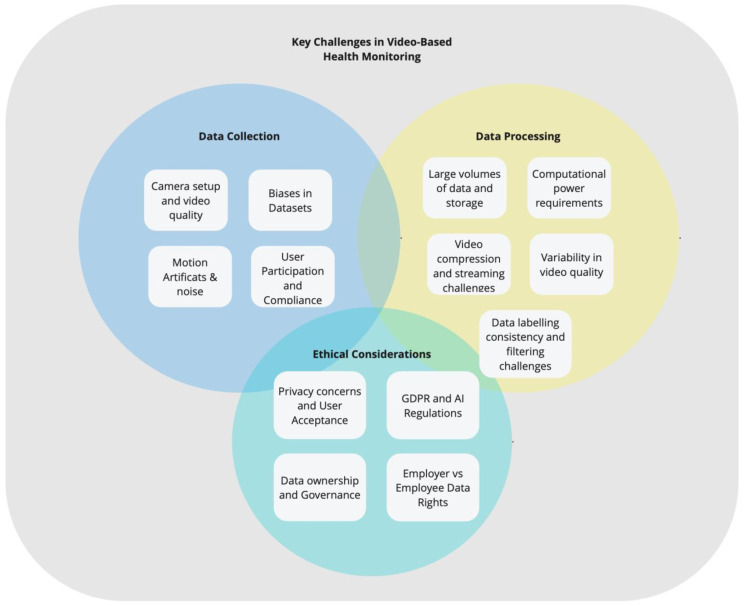
Key Challenges in Video-Based Health Monitoring.

Despite the challenges associated with data collection (Figure 2), processing, and privacy regulations, video-based health monitoring continues to demonstrate strong potential for real-world healthcare applications. As this technology evolves, it is reshaping healthcare by unlocking new opportunities in patient care, disease management, and clinical research, making continuous, real-time physiological and behavioral monitoring more feasible and impactful in both clinical and everyday settings. By leveraging the widespread availability of cameras and computing power, this approach offers a non-invasive and cost-effective alternative to traditional monitoring methods, bridging gaps in healthcare accessibility and supporting proactive, data-driven interventions.

The capability to remotely monitor health-related variables is particularly beneficial for disease management, early risk detection, and continuous health status assessment. Video-based systems can track physiological trends over time, ensuring timely interventions for conditions such as chronic stress, cardiovascular health monitoring, and mental health risk detection. Additionally, they enable personalized health insights by monitoring progress, adherence, and the execution of prescribed exercises, allowing healthcare providers to tailor interventions based on real-time data. This not only increases the efficiency of healthcare delivery by reducing the burden on in-person consultations and hospital visits, but also enhances patient autonomy and accessibility, providing individuals with high-quality care from the comfort of their daily environments.

Furthermore, the ability to detect subtle physiological and behavioral changes through video analysis enhances preventive care strategies. The delivery of data, along with the user’s ability to share these data with designated parties, also offers significant potential for the early detection of health issues before they become critical. By leveraging AI-driven pattern recognition, healthcare professionals can identify deviations from baseline behaviors, allowing for timely medical evaluations. Subtle changes in behavior, mobility, or facial expressions can signal underlying conditions that may require immediate medical attention. Early intervention, guided by these indicators, can significantly improve patient outcomes.

A particularly promising application lies in mental health assessment, where continuous video-based monitoring can complement traditional diagnostic methods. With the integration of emotion recognition technologies, video monitoring can play a vital role in mental health assessment. By integrating physiological markers such as facial activity and blinking rate with emotional state analysis, these systems can provide a more comprehensive view of mental well-being. By analyzing facial cues and body language, healthcare providers can gauge emotional states, providing valuable data that can inform treatment plans for conditions such as depression, anxiety, and stress.

The wealth of data collected through video monitoring can be analyzed to yield insights into health trends, treatment effectiveness, and lifestyle factors that influence well-being. These data can drive personalized healthcare, inform public health strategies, and enhance the overall quality of healthcare services. Moreover, integrating AI-powered analytics with these data can uncover previously unnoticed patterns in health deterioration, further strengthening its role in predictive healthcare.

Delving deeper into the vast possibilities, in clinical research, video monitoring can provide an objective and consistent means of measuring and recording study endpoints. This can improve the quality of data collection, reduce the reliance on self-reported measures, and potentially accelerate the pace of clinical trials. With automated data capture and AI-assisted video analysis, researchers can ensure greater accuracy and standardization of health metrics across diverse study populations. For medical professionals, video-based monitoring systems can serve as educational tools, allowing for the review and analysis of interactions, procedures, and treatment outcomes. This can enhance the training process and improve the skill set of healthcare workers.

## 6. Conclusions and Future Considerations

The exploration of video-based health monitoring during this pilot study has culminated in a nuanced appreciation of its potential, set against a backdrop of technological advancements and healthcare imperatives. The findings reveal a distinct path for future work in this field, positioning the study as a launching point from which subsequent research can advance.

As we draw conclusions from our study, it becomes evident that the integration of video-based health monitoring can substantially enhance worker engagement, compliance, and satisfaction. The capability for healthcare providers to monitor and respond to worker needs in real time can transform the current paradigms of healthcare delivery, creating a more dynamic, responsive, and user-centered system.

The potential of video monitoring extends beyond traditional care into the realms of medical research and clinical trials. The high-fidelity data it provides can refine research methodologies and contribute to a richer, more accurate understanding of health conditions and the impact of medical interventions.

Looking ahead, the focus should be on refining the technologies and methodologies used in video-based monitoring. Future studies must aim to address the limitations noted in our pilot, particularly the need for robust, unobtrusive systems that respect user privacy while providing accurate health data. As AI and machine learning technologies advance, so too should the algorithms that drive video-based health assessments, evolving to handle diverse datasets with sophistication and sensitivity to context.

The ethical aspects related to privacy and data security require vigilant navigation. Future efforts should focus on developing protocols that protect participant data, ensuring that the advantages of video-based monitoring are not achieved at the expense of personal privacy. To address concerns surrounding implicit coercion in workplace monitoring, future implementations should address the same principles incorporated in this research, namely enhanced informed consent protocols, emphasizing voluntary participation, the right to withdraw at any time without consequences, and full disclosure of data usage policies. Ensuring transparency in data lifecycle management, participant ownership, and access rights are crucial in maintaining trust in health monitoring systems.

Furthermore, adopting third-party data governance models—where participant data are managed by an independent entity rather than the employer—can provide an additional layer of protection against potential conflicts of interest. Employers should only receive aggregated, team-level insights (for groups larger than 10 employees) rather than individual data, ensuring that workplace monitoring does not become a tool for targeting specific employees but instead serves as a health-focused, employee-first initiative.

A worker-centric approach, grounded in data transparency, privacy protection, and ethical oversight, is essential to ensuring that video-based health monitoring remains a tool for supporting employees rather than evaluating them.

Video-based health monitoring stands at the threshold of a new era in healthcare. The convergence of technology, medicine, and patient-centric care models beckons a future where health monitoring is seamlessly integrated into the fabric of daily life, empowering individuals and healthcare providers alike. It is incumbent upon the research community to continue pushing the boundaries of what is possible, fostering innovations that will underpin the healthcare solutions of tomorrow.

## Figures and Tables

**Table 1:** Advantages and disadvantages of monitoring systems.

Monitoring Method	Advantages	Disadvantages	Effective/Real Impact
Video-based (AI-enhanced)	Non-invasive, does not require wearables, real-time tracking of facial features	Sensitive to lighting conditions, requires computational power, potential privacy concerns	Provides continuous, unobtrusive physiological monitoring, enabling early stress detection, fatigue assessment, and workplace health insights without requiring physical devices. Effective in scenarios where wearables may be impractical.
Wearable devices (e.g., smartwatches, biosensors)	High accuracy, continuous physiological monitoring	Requires user compliance, intrusive for some users, battery life limitations	Highly effective for real-time tracking of heart rate and activity levels, particularly for mobile users, athletes, and clinical monitoring, but is highly dependent on the user.
Clinical methods (e.g., ECG, polysomnography)	Gold standard for accuracy	Expensive, requires physical presence, not scalable for daily monitoring	Most reliable for diagnosing cardiovascular conditions and sleep disorders, but impractical for continuous long-term monitoring outside a clinical setting.

## Data Availability

Data are contained within the article.
